# Pre-treatment of eucalypts biomass towards enzymatic saccharification

**DOI:** 10.1186/1753-6561-5-S7-P116

**Published:** 2011-09-13

**Authors:** Washington Luiz Magalhães, Cristiane Helm, Patricia Silva, Edson Lima, Kleber Hoffman, Amanda Higa, Tielidy Lima

**Affiliations:** 1 Embrapa Forestry, Brazil; 2 UFPR, Brazil

## Background

There are a few possible ways to produce ethanol from lignocellulosic biomass, for instance, thermochemical and acid hydrolysis. However, enzymatic hydrolysis of carbohydrates is considered the greenest process for saccharification, followed by sugar fermentation into ethanol.

The main challenge of the enzymatic saccharification process is that cellulose is not exposed to the enzyme action in the lignin matrix. The cellulose molecules are arranged in semi-crystalline nanofibrils immersed in lignin matrix with hemicelluloses (polyoses) and extractives between them acting as coupling agents. These nanofibrils are placed together to form helical microfibrils inside the cell wall. Thus, a pre-treatment is necessary to make room for the enzymes to reach the cellulose fibril surfaces in order for the whole process to become economically feasible.

There are many pre-treatments proposed in specialized literature, but their efficiencies are dependent on the biomass composition [1-4]. Moreover, these treatments have to address some constraints such as the recyclability of the chemicals used, low consumption of energy, and sustainability concerns.

We devised a future possibility of a cellulose pulp mill to be transformed into a biorefinary, where besides cellulose pulp, ethanol could also be produced. In the Brazilian pulp mill industry, the process most commonly used is the Kraft process, so the digestion with green and white liquors can be adapted for pre-treatment towards enzymatic saccharification. Also, the industry had already tackled recycling of the black liquor – obtained after wood chips digestion –, recuperating thermal energy by burning lignin and recovering the green liquor.

This work is part of our research to evaluate some modifications on the green liquor digestion towards enzymatic saccharification. We evaluated efficiencies of some pre-treatments with green liquor through enzymatic hydrolysis for holocellulose saccharification.

## Methods

A wood log from a 7-year old tree of *Eucalyptus benthamii* was cut into discs. They were cut into 30° wedges and were then grounded using a Willey mill and sifted to be between 20 and 40 Tyler mesh.

Composition of the *E. benthamii* wood obtained by NBR standards was: 3% of total extractives; 28.4% of Klason lignin; 0.03% of ash and 3.5 of syringyl:guaiacyl molar ratio.

Table 1 shows the characterization of the green liquor acquired from Iguaçu Celulose Papel S.A.

**Table 1:** Characterization of the industrial green liquor used in this work.

Analysis of green liquor	Content (g.L^-1^)
Content of sodium sulfide (X) in Na_2_S	14.59
Content of sodium sulfite (Y) in Na_2_SO_3_	13.9
Content of sodium thiosulfate (Z) in Na_2_S_2_O_3_	3.16
Total alkali (concentration of alkaline constituents: OH^-^+HS^-^+HCO_3_^-^) expressed in NaOH	114.1
Active alkali (sum of concentrations of hydroxyl and hydro-sulfite ions including ions formed by hydrolysis of sulfides: OH^-^+HS^-^) expressed in NaOH	43.3
Effective alkali (concentration of hydroxyl ions including those formed from sulfides by hydrolysis: OH-) expressed in NaOH	32.5
Sulfidity	33.7 %

The pre-treatment conditions used in this study were two levels of temperature – 120 and 180 ^o^C – and three levels of reaction time – 1, 2, and 3 hours. Cylindrical stainless steel reactors (Parr, USA) with volume capacity of 50 mL were used. The reactors were heated in an aluminum dry block furnace (Marconi, Brazil) with a temperature and time controller. The ratio of solid wood and green liquor was 1:4 (g.mL^-1^). Typical values were 4 g of dry biomass in 16 mL of green liquor per reactor.

The pre-treated biomass was digested using Falcon tubes with enzymes in a buffer solution of sodium citrate (pH of 5.0) in an incubator (Tecnal TE-420, Brazil) at 50 °C and 250 rpm during 24 hours. The enzymes used were cellobiase from *Aspergillus niger* (Novozyme 188) and cellulase from *Trichoderma reesi* (ATCC 26921), acquired from Sigma Aldrich, USA. Typically, the proportion was 0.2 g of dry biomass, 40 ml of buffer solution, 46 mL of cellobiase and 90 mL of cellulase (both used as received). After saccharification, the hydrolyzate was filtered and the reducing sugars were quantified through the dinitrosalicylic acid (DNS) method.

## Results

Table 2 shows the solid mass loss after treatment, the total reducing sugar after enzymatic hydrolysis, and the global yield of saccharification.

**Table 2:** Mass loss after pre-treatment, total reducing sugar released after enzymatic digestion, and the yield of conversion based on holocellulose content in the untreated biomass.

Treatment	Mass loss (%)	Total reducing sugar (kg.kg^-1^ of treated biomass)	Yield of sugar conversion based on holocellulose of untreated biomass (%)
untreated	0.0	0.04	5.6
120°C, 1h	26.5	0.10	10.6
120°C, 2h	26.4	0.13	14.1
120°C, 3h	34.3	0.16	15.6
180°C, 1h	47,3	0.92	70.7
180°C, 2h	52.5	0.78	53.9
180°C, 3h	50.8	0.91	65.7

As can readily be seen on Table 2, temperature plays a crucial role in exposing cellulose for enzymatic hydrolysis, as does the length of cooking. For lowest temperatures, (120 °C) increasing time causes increased sugar release and conversion yield. On the other hand, for the highest temperature level (180 °C), the time of cooking plays a more complex role. The treatment at 180 °C after 1 hour reaches the highest sugar conversion based on holocellulose content of untreated biomass.

The above results can be explained by the following suppositions. Increasing treatment time to 2 and 3 hours results in hemicellulose and amorphous cellulose losses, which decreased conversions yield. However, when the treatment time was increased to 3 hours, besides amorphous holocellulose loss, there was also the occurrence of crystalline modification of part of the crystalline cellulose type I into amorphous and cellulose type II. Both amorphous and crystalline type II cellulose are enzymatic digested easier than crystalline type I cellulose. This could explain why sugar conversion yield after 2 hours of treatment at 180 ^o^C are smaller than after 3 hours. XRD and chemical characterizations of the pre-treated biomass have to be done in order to corroborate with the above suppositions.

## Conclusion

Simple modifications of the Kraft process can be considered as an alternative route for pre-treatment of woody biomass towards enzymatic saccharification.
